# Chemical Composition and Antimicrobial Activity of the Essential Oil of Kumquat (*Fortunella crassifolia* Swingle) Peel

**DOI:** 10.3390/ijms13033382

**Published:** 2012-03-12

**Authors:** Yong-Wei Wang, Wei-Cai Zeng, Pei-Yu Xu, Ya-Jia Lan, Rui-Xue Zhu, Kai Zhong, Yi-Na Huang, Hong Gao

**Affiliations:** 1 Department of Public Health, Hua Xi Medicinal Center of Sichuan University, Chengdu 610041, China; E-Mails: wangyongwei_19801025@yahoo.com.cn (Y.-W.W.); xpy9929@163.com (P.-Y.X.); gh@scu.edu.cn (Y.-J.L.); 2 West China Fourth Hospital & Occupational Diseases Hospital, Hua Xi Medicinal Center of Sichuan University, Chengdu 610041, China; 3 College of Light Industry, Textile and Food Engineering, Sichuan University, Chengdu 610065, China; E-Mails: scuzwc@163.com (W.-C.Z.); zhu_ruixue@126.com (R.-X.Z.); eric211@163.com (K.Z.)

**Keywords:** *Fortunella crassifolia*, essential oil, chemical composition, food-borne bacteria, antimicrobial activity

## Abstract

The aim of this study was to determine the main constituents of the essential oil isolated from *Fortunella crassifolia* Swingle peel by hydro-distillation, and to test the efficacy of the essential oil on antimicrobial activity. Twenty-five components, representing 92.36% of the total oil, were identified by GC-MS analysis. The essential oil showed potent antimicrobial activity against both Gram-negative (*E. coli* and *S. typhimurium*) and Gram-positive (*S. aureus*, *B. cereus*, *B. subtilis*, *L. bulgaricus*, and *B. laterosporus*) bacteria, together with a remarkable antifungal activity against *C. albicans*. In a food model of beef extract, the essential oil was observed to possess an effective capacity to control the total counts of viable bacteria. Furthermore, the essential oil showed strongly detrimental effects on the growth and morphological structure of the tested bacteria. It was suggested that the essential oil from *Fortunella crassifolia* Swingle peel might be used as a natural food preservative against bacteria or fungus in the food industry.

## 1. Introduction

Kumquats (*Fortunella* spp.) belong to the *Citrus* genus are fruit, usually eaten raw as a whole fruit together with the peel, excluding the seeds. The peel is sweet and edible with a typical aroma due to the presence of flavonoids and terpenoids [1]. Kumquats are also an excellent source of nutrients and phytochemicals, including ascorbic acid, carotenoids, flavonoids and essential oils. Some *Fortunella* plants, such as *Fortunella japonica* Swingle, *Fortunella margarita* (Lour.) Swingle and *Fortunella crassifolia* Swingle are commonly cultivated in the southern region of China. *F. crassifolia*, known as jingdan or meiwa kumquat, has a typical citrus character. As with other citrus fruits, *F. crassifolia* can be candied, prepared as marmalade, added to fruit salad and preserved as a whole in sugar syrup in food industry.

It is well known that *F. crassifolia* is rich in flavonoids. Previously, 3′,5′-di-*C*-β-glucopyranosylphloretin, a characteristic flavonoid, was well-studied in the genus *Fortunella* [2]. Recently, the antioxidant activity of *F. crassifolia* has been reported [3]. However, studies on the chemical composition and biological activities of *F. crassifolia* peel oils are limited. From a dietary viewpoint, essential oils represent added value for kumquat fruit because, in addition to their contribution to the flavor, they play an important role in human health, as with other non-nutritive phytochemicals such as polyphenols and flavonoids [4].

The *Citrus* essential oils have various functional properties, such as an attractive aroma, a repellant agent against insects and animals, and antioxidant activities. A previous study has reported the antimicrobial action of citrus oils [5]. Meanwhile, citrus oils are not only available to the food industry, but are also generally recognized as safe and have been found to be inhibitory, in both oil and vapor form, against a range of both Gram-positive and Gram-negative bacteria [6]. Moreover, there are a large number of studies on plant essential oils regarding their antimicrobial properties in order to develop a source of antimicrobial ingredients for the food industry [7–9].

To our knowledge, there are no reports in the literature concerning the antimicrobial activity of the essential oil from *F. crassifolia* until now. The aim of this study was therefore to determine the main constituents of the essential oil of *F. crassifolia* peel, together with an evaluation of its antimicrobial activity.

## 2. Results and Discussion

### 2.1. Chemical Composition in the Essential Oil

Essential oil was obtained from the fresh peel of *F. crassifolia* by hydro-distillation and subjected to gas chromatography-mass spectroscopy (GC-MS). Twenty-five components, representing 92.36% of the total oil, were identified by GC-MS analysis. These compounds identified by GC-MS analysis, their retention indices and relative area percentages are shown in Table 1. A total of twelve terpenoid hydrocarbons amounting to a relative 85.36% of the essential oil were identified. Terpenoid hydrocarbons were the characteristic and the most abundant constituents, as in other citrus fruits. The amount of the major compound, limonene, in the essential oil obtained from *F. crassifolia* peel is high (74.79%), followed by myrcene (7.11%), camphene (1.43%), α-selinene (0.7%), α-pinene (0.34%), 3,4-dimethyl styrene (0.32%), and β-elemene (0.29%). *p*-Cymene, γ-terpinene, 2,7-dimethyl-1,6-dioctadione, α-copaene and bicyclgermacrene occurred at <0.1% in the essential oil. A previous report showed that the major chemical component of citrus oils is limonene, ranging from 32 to 98%, with sweet orange containing 68–98%, lemon 45–76% and bergamot 32–45% [6,10]. In another study on the characteristic odor components in the essential oil from *F. japonica* peel, limonene was the most dominant component, amounting to 93.73% [11]. The limonene content of *F. crassifolia* peel oil was comparatively less than that reported for *F. japonica* peel oil. The α-pinene content of the essential oil was similar to that reported for *F. japonica* peel oil [11]. However, the contents of myrcene, camphene and α-selinene were found to be higher than those reported for *F. japonica* peel oil [4,11]. The essential oil contained five alcohols (3.31%): linalool (0.30%), carveol (0.13%), *p*-mentha-2,8-dien-1-ol (1.00%), *p*-mentha-1,5-dien-8-ol (1.65%) and spathulenol (0.22%). The alcohol contents of the oil presented here were much higher than those of *F. japonica* [4,11]. Ketones constituted 1.80%, where carvone and piperitenone accounted for 1.58% and 0.22%, respectively. The amount of esters was detected to be 1.72% in this oil, with the five main compounds being: octyl acetate (0.62%), *trans*-myrtanyl acetate (0.62%), perillyl acetate (0.22%), *cis*-myrtanyl acetate (0.15%), and isopropyl cinnamate (0.11%). Perillaldehyde (0.18%) was found to be the only aldehyde compound. The amounts of ketones, esters and aldehydes were low, as with other *Fortunella* species oils [4,11].

### 2.2. Antimicrobial Activity

The essential oil from *F. crassifolia* peel was evaluated for antimicrobial activity. These activities were qualitatively and quantitatively assessed according to the presence or absence of inhibition zones, zone diameters, MIC and MBC values. According to the results given in Table 2, the essential oil showed antibacterial activity against food pathogenic strains of Gram negative (*E. coli* and *S. typhimurium*) and Gram positive (*S. aureus* and *B. cereus*) bacteria. It was found to be active against all of the tested bacterial strains in the range of inhibition zones of 13.7–20.8 mm. It also showed a remarkable antifungal activity against *C. albicans* with an inhibition zone of 13.7 ± 0.75 mm, MIC value of 70.0 μg/mL and MBC value of 72.5 μg/mL, respectively. It is obvious that the essential oil has both a strong effect and a broad spectrum on antimicrobial activity.

As emphasized elsewhere, Gram-positive bacteria are more sensitive to plant oils than Gram-negative bacteria, which may be due to relatively impermeable outer membranes that surround Gram-negative bacteria. This differential sensitivity has been observed when using citrus oils/components *in vitro* [12–14]. In our study, the MIC values of the essential oil for Gram-negative bacteria ranged from 50 to 70 μg/mL. For Gram-positive bacteria, the values were 37.5 to 67.5 μg/mL. The MBC values for Gram-negative bacteria ranged from 52.5 to 72.5 μg/mL, and 40–70 μg/mL for Gram-positive bacteria. Although the activity of the oil varied with its concentration and kind of bacteria, these results showed that *F. crassifolia* essential oil did not possess any selective antimicrobial activity on the basis of the cell wall differences of Gram-negative or Gram-positive bacteria. Generally, the concentrations of the oil were about 100 times higher than those of the positive control (Gentamicin). This difference between concentrations of the essential oil and the standard antibiotic can be explained in terms of the fact that the active components in the oil comprise only a fraction of the oil used and the concentration of the active components could be much lower than the standard antibiotics used [15].

Essential oils are a complex of volatile compounds and may exhibit the potent antimicrobial activity by a single major compound or by the synergistic or antagonistic effect of various compounds [16]. Research into the antimicrobial effects of monoterpenes suggests they diffuse into cells and damage cell membrane structures [17]. Limonene and α-pinene, which were found to be abundant in the oil in this study, have been reported to show antifungal activity [18,19]. However, a previous study has reported that lemon, sweet orange and bergamot and their components, linalool and citral (but not limonene) showed antimicrobial effects both in oil and vapor form against *Campylobacter jejuni*, *E. coli* O157, *L. monocytogenes*, *B. cereus* and *S. aureus* [6]. The previous studies suggested that limonene showed a weak antibacterial activity when compared with its antifungal activity. The antimicrobial activity of the essential oil could be enhanced by the presence of bioactive alcohols, such as linalool and carveol. Linalool, a monoterpene alcohol in this oil, was known as a potent antimicrobial compound and was reported to show a wide range of antibacterial and antifungal activity [6,20,21]. Carveol, although a minor component in the oil, has been reported to possess a wide range and high levels of antimicrobial activity [22]. The major ketone compound, carvone, which occurs at levels of 1.58% in the oil, has been reported to show potent antimicrobial activity [23]. Further studies are underway for the antimicrobial assessment of the main compounds in the essential oil and a toxicological evaluation for the safe usage of the oil in food products.

### 2.3. Antibacterial Activity the Essential Oil in a Food Model Media

**Figure 1:**
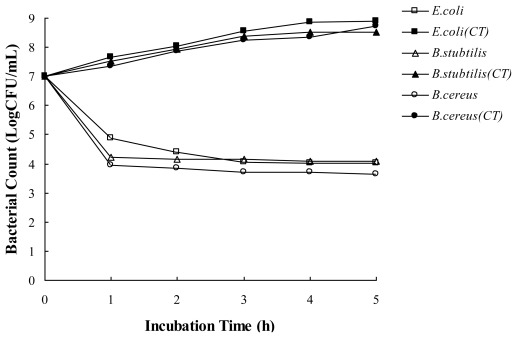
Effect of the essential oil from *Fortunella crassifolia* Swingle peel on viability of the tested bacteria in beef extract media. The concentration of the essential oil was set at MIC concentration for the tested bacteria. CT: control without treatment.

The antibacterial activity of the essential oil in a food model media was evaluated through investigating the effect of the essential oil on viable counts of bacteria (*E. coli*, *B. subtilis* and *B. cereus*) in meat-based model media (autoclaved beef extract, BE, 3% in deionized water). As shown in Figure 1, the bacteria without the essential oils grew slowly over a 5 h period. However, bacteria exposed to the essential oil showed a steep decline in CFU numbers during the first 1 h, with bacteria counts then remaining stable until the end of the assay. The exposure time of the essential oil for complete inhibition of cell viability of all the tested bacteria was found to be 1 h, when the essential oil exerted its maximum bactericidal activity at MIC level. Moreover, *B. cereus* was found to be the most sensitive; the oil exerted maximum bacterial activity against *B. cereus* with the lowest concentration. Therefore, the MIC of the oil had a severe effect on the cell viability of the tested bacteria.

### 2.4. Observation with Transmission Electron Microscope (TEM)

**Figure 2:**
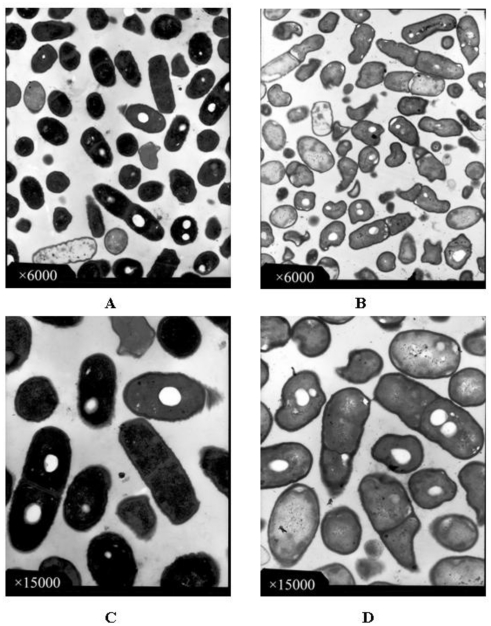
Transmission electron microscope diagram of *B. subtilis* in the absence (**A,C**) and presence (**B,D**) of the essential oil from *Fortunella crassifolia* Swingle peel.

TEM photomicrographs of *B. subtilis* in the presence or absence of the essential oil are shown in Figure 2. *B. subtilis* cells in the control group showed normal morphology, regular outlined cell walls and cytoplasmic membranes lying close to the cell walls. Cytoplasm regularly filled the entire cells (Figure 2A,C). After treated with the essential oil at MIC level, the cells were damaged seriously (Figure 2B) compared with the cells in control. The cells with incomplete cytoplasm were distributed everywhere within the field of view. According to Figure 2D (at greater magnification), the cell walls and membranes of the *B. subtilis* were partially disintegrated, causing the outflow of electron-translucent cytoplasm, and the cells became empty. The localized separation of cell membranes from cell walls could also be observed from the photomicrographs. The essential oil showed an obvious influence on the morphological structure of *B. subtilis*. Previous findings suggested that essential oils can alter cell fluidity/permeability and functions by entering between the fatty acyl chains making up membrane lipid bilayers and disrupting the lipid packing [17,24]. The loss of the differential permeability characteristics of the cytoplasmic membrane is frequently identified as the cause of cell death.

## 3. Materials and Methods

### 3.1. Isolation of the Essential Oil

Fresh *F. crassifolia* fruit was purchased from a local market in Chengdu, Sichuan province of China, in December 2010. The plant was initially identified by morphological features and the database present in the Department of Biology, Sichuan University. A voucher specimen (No. 20091206) was dried and preserved at the Key Laboratory of Food Science and Technology of Sichuan Province, Sichuan University.

The individual fruits were washed with water and then separated into peel and flesh by hand. The fresh peelings (250 g) were homogenized with a mixer, followed by hydro-distillation using a Clevenger-type apparatus for 4 h. The essential oil was collected over water, dried over anhydrous sodium sulfate and stored at 4 γC until analyzed. The oil yield was about 2% (5 g/250 g of fresh peel).

### 3.2. Gas Chromatography-Mass Spectroscopy (GC-MS) Analysis

The sample of essential oil (5 μL) was diluted in *n*-hexane (0.5 mL) and analyzed with the Thermo GC-MSD (Trace DSQ II, Thermo Fisher Corporation, USA) apparatus equipped with a TR-5 capillary column (30 m × 0.25 mm internal diameter, 0.25 μm film thickness). Helium (1.0 mL/min) was used as a carrier gas. The injector and the transfer line were kept at 250 γC and 280 γC, respectively. The column was maintained at 60 γC for 4 min and then programmed to rise to 240 γC at 4 γC/min and held for 15 min at 240 γC. The MS was operated in the EI mode at 70 eV and in the *m*/*z* range 40–500. Retention indices of the separated compounds on the TR-5 capillary column were determined on the basis of a homologous series of *n*-alkanes (C_9_–C_27_). The compounds of essential oil were identified on the basis of comparison of their retention indices and mass spectra with published data [25] and computer matching with National Institute of Standards and Technology (NIST, 3.0) libraries provided with a computer controlling the GC-MS system. The relative proportions of the essential oil constituents were expressed as percentages obtained by peak area normalization, and all relative response factors were taken as one.

### 3.3. Test Microorganisms

The tested microorganisms contained Gram-negative (*Escherichia coli* ATCC 25922, *Salmonella typhimurium* ATCC 14028, Gram-positive (*Staphylococcus aureus* ATCC 25923, *Bacillus subtilis* ATCC 21216, *Bacillus cereus* ATCC 10231, *Lactobacillus bulgaricus* ATCC 11842, *Bacillus laterosporus* ATCC 64) bacteria, and fungus (*Candida albicans* ATCC 50013). All of the microorganisms were provided by the Key Laboratory of Food Science and Technology of Sichuan Province, Sichuan University, China. All the strains were maintained on nutrient agar at 4 γC and were subcultured every month in our laboratory.

### 3.4. Antimicrobial Activity

A nutrient agar culture medium was used to culture bacteria. A medium of potatoes was used to culture fungus. Antimicrobial activity of the essential oil was determined by the Oxford plate method [26]. Briefly, bacterial cultures were diluted to obtain a bacterial suspension of 10^8^ CFU/mL with distilled water. Petri plates containing 20 mL of nutrient agar were inoculated with 200 μL of bacterial culture and allowed to dry in a sterile chamber. The Oxford plates (6 mm in diameter) were impregnated with 100 μL of 50 mg/mL essential oil (dissolved in 50% methanol, distilled with sterile water) and placed on the inoculated agar. The 50% methanol was also used as the negative control. Gentamicin (50 μg/disc) was used as the positive control. The inoculated plates were incubated at 37 γC for 24 h. The antimicrobial activity was evaluated by measuring the zone of inhibition against the test organisms.

The minimum inhibitory concentration (MIC) of the essential oil against the test bacterial strains was determined by the micro-well dilution method [27]. All of the microorganisms were prepared for 24 h and the suspensions were adjusted to 10^8^ CFU/mL. The essential oil was dissolved in 50% methanol and serial two-fold dilutions of the oil were prepared in a 96-well plate, ranging from 2.5 μg/mL to 960 μg/mL. The MIC was defined as the lowest concentration of the essential oil at which the microorganism did not demonstrate visible growth. Microorganism growth was indicated by turbidity.

Minimum bactericidal concentration (MBC) was determined by sub-culturing 10 μL of the MIC test solutions on nutrient agar plate at 37 γC for 24 h. The highest dilution that yielded no bacterial growth was taken as MBC.

### 3.5. Antibacterial Activity of the Essential Oil in a Food Model

To investigate the antibacterial activity in meat-based model media, experiments were undertaken with autoclaved beef extract (BE, 3% in deionized water). BE media were adjusted to pH 7.2 [28]. For viable counts, tubes containing 10 mL of BE media and a bacterial suspension (approximately 10^7^ CFU/mL) of *E. coli*, *B. subtilis* and *B. cereus* were inoculated with MIC of the essential oil, and kept at 37 γC. Samples for viable counts were taken out at 0, 1, 2, 3, 4 and 5 h time intervals. The viable plate counts were monitored as follows: 0.1 mL sample of each treatment was diluted and spread on the surface of nutrient agar. The colonies were counted after 24 h of incubation at 37 γC. The controls were inoculated without essential oil for each bacterial strain under the same experimental conditions described above.

### 3.6. Observation with Transmission Electron Microscope (TEM)

TEM observation for *B. subtilis* was determined by our previous method [8]. Nutrient broth containing *B. subtilis* (cultured for 24 h) was centrifuged at 5000 rpm at 25 γC for 10 min, and the precipitate was washed three times with PBS (0.1 M, pH 7.4). An amount of glutaraldehyde (0.5%) was added to the precipitate and maintained at 15 min at 4 γC. *B. subtilis* cells were collected by 20 min centrifugation (15,000 rpm, 4 γC). After dealing with 3% glutaraldehyde, 1% osmium tetroxide, acetone and epoxy resins, the sample was cut into thin sections using a microtome (Ultracut-E, Reichert-Jung, Austria). Then the sample was observed by TEM (H-600IV, Hitachi, Japan).

### 3.7. Statistical Analysis

All experiments were performed in triplicate. The data were recorded as means ± standard deviations and analyzed with SPSS (version 12.0 for Windows, SPSS Inc., 223 South Wacker Drive, Chicago, USA). Differences were considered significant at *P* < 0.05.

## 4. Conclusion

In summary, the composition of the essential oil of *F. crassifolia* peel has been analyzed by GC-MS and its antimicrobial activity investigated. The essential oil contained terpenoid hydrocarbons (85.42%), alcohols (3.3%), ketones (1.8%), esters (1.72%) and aldehydes (0.18%). The essential oil showed a wide range of antimicrobial activity against both bacteria (Gram negative and Gram positive) and fungus (*C. albicans*). It is important to note that the essential oil was effective on inhibiting food-borne and pathogen bacteria, particularly *B. cereus* among the tested bacteria strains. In a meat-based food model, the essential oil showed an obvious effect on the cell viability of the tested bacteria. Moreover, TEM analysis showed the change of external morphological features of *B. subtilis* treated by the essential oil. These results suggested the potential use of the essential oil in the food industry for the preservation of foodstuffs against bacteria or fungus, and for increasing the shelf life of foodstuffs.

## Figures and Tables

**Table 1:** The main chemical components of the essential oil in the fresh *Fortunella crassifolia* Swingle peel.

NO.	RI a	Compound	Identification b	(%) c
1	936	α-Pinene	MS, RI	0.34
2	955	Camphene	MS, RI	1.43
3	993	Myrcene	MS, RI	7.11
4	1021	*p*-Cymene	MS, RI	0.01
5	1035	Limonene	MS, RI	74.79
6	1064	γ-Terpinene	MS, RI	0.09
7	1072	2,7-Dimethyl-1,6-octadione	MS	0.02
8	1091	3,4-Dimethyl styrene	MS	0.32
9	1097	Linalool	MS, RI	0.30
10	1138	Carveol	MS, RI	0.13
11	1146	*p*-Mentha-2,8-dien-1-ol	MS, RI	1.00
12	1164	*p*-Mentha-1,5-dien-8-ol	MS, RI	1.65
13	1209	Octyl acetate	MS, RI	0.62
14	1245	Carvone	MS, RI	1.58
15	1365	Piperitenone	MS	0.22
16	1371	Perillaldehyde	MS	0.18
17	1376	α-Copaene	MS, RI	0.18
18	1381	*cis*-Myrtanyl acetate	MS, RI	0.15
19	1383	*trans*-Myrtanyl acetate	MS, RI	0.62
20	1392	β-Elemene	MS, RI	0.29
21	1459	Perillyl acetate	MS	0.22
22	1498	Bicyclogermacrene	MS, RI	0.08
23	1506	α-Selinene	MS, RI	0.70
24	1578	Spathulenol	MS, RI	0.22
25	1861	Isopropyl cinnamate	MS	0.11
Total				92.36

a RI, retention indices as determined on TR-5 capillary column using homologous series of *n*-alkanes.

b Methods of identification: MS, by comparison of the mass spectrum with those of the computer mass libraries; RI, by comparison of RI with those from the literature.

c (%), relative percentage obtained from peak area.

**Table 2:** Antimicrobial activity of the essential oil from *Fortunella crassifolia* Swingle peel.

		Inhibition zone (mm)		Essential oil (μg/mL)	
		
Microorganisms	Source	Essential oil a	Gentamicin b	MIC c	MBC d
Gram negative					
*E. coli*	ATCC 25922	18.0 ± 1.29	26.0 ± 0.10	50.0	52.5
*S. typhimurium*	ATCC 14028	13.2 ± 0.37	23.3 ± 0.47	70.0	72.5
Gram positive					
*S. aureus*	ATCC 25923	14.8 ± 0.69	27.3 ± 0.47	65.0	67.5
*B. subtilis*	ATCC 21216	20.2 ± 0.37	23.3 ± 0.47	47.5	50.0
*B. cereus*	ATCC 10231	20.8 ± 0.69	22.0 ± 0.40	37.5	40.0
*L. bulgaricus*	ATCC 11842	18.2 ± 1.07	24.7 ± 0.47	67.5	70.0
*B. laterosporus*	ATCC 64	18.3 ± 0.94	20.3 ± 0.45	67.5	70.0
Fungus					
*C. albicans*	ATCC 50013	13.7 ± 0.75	27.3 ± 0.47	70.0	72.5

a The concentration of essential oil was 5 mg/disc.

b The concentration of gentamicin was 50 μg/disc.

c Minimum inhibitory concentration.

d Minimum bactericidal concentration.
